# Prospective Associations of Behavioural and Organisational Factors with Initial Nursing Diagnosis Use: A Longitudinal Study

**DOI:** 10.3390/nursrep16090321

**Published:** 2026-09-04

**Authors:** Mattia Bozzetti, Ilaria Marcomini, Roberta Pendoni, Alberto Silla, Miriam Mariani, Gianmario Pedretti, Alessio Conti, Alessio Lo Cascio, Greta Ghizzardi, Daniele Napolitano

**Affiliations:** 1 Direction of Health Professions, ASST Cremona, 26100 Cremona, Italy; alberto.silla@asst-cremona.it (A.S.); miriam.mariani@asst-cremona.it (M.M.); gianmario.pedretti@asst-cremona.it (G.P.); 2 Center for Nursing Research and Innovation, Faculty of Medicine and Surgery, Vita-Salute San Raffaele University, 20132 Milan, Italy; marcomini.ilaria@unisr.it; 3 Research and Innovation Unit for Health Professions, San Luigi Gonzaga University Hospital, 10043 Orbassano, Italy; alessio.conti@unito.it; 4 Department of Clinical and Biological Sciences, University of Turin, 10043 Turin, Italy; 5 Clinical Trial Unit, La Maddalena Cancer Center, 90146 Palermo, Italy; locascio.alessio@lamaddalenanet.it; 6 Healthcare Professions Directorate, ASST Lodi, 26900 Lodi, Italy; greta.ghizzardi@asst-lodi.it; 7 SITRA–Scientific Direction, Fondazione Policlinico Gemelli IRCSS, 00168 Rome, Italy; daniele.napolitano@policlinicogemelli.it

**Keywords:** nursing diagnosis, standardised nursing terminologies, nursing documentation, work environment

## Abstract

**Background/Objectives:** Nursing diagnoses (NDs) support clinical reasoning, individualised care planning, and the visibility of nursing practice, yet their implementation remains inconsistent. This study prospectively examined whether TPB-informed beliefs, attitudes towards NDs, behavioural intention, and the nursing practice environment were associated with initial ND use and subsequent documentation intensity. **Methods:** A prospective observational cohort study was conducted across 14 adult inpatient units within a public healthcare organisation in Northern Italy. Baseline questionnaire data were linked with electronic nursing records, workforce data, and ward-level administrative data. Initial use was defined as documentation of at least one ND during follow-up. **Results:** The primary cohort included 144 nurses, of whom 122 (84.7%) documented at least one ND. Greater work exposure was associated with initial use (OR = 1.71, 95% CI [1.12, 2.60]). After additional adjustment for the overall PES-NWI, behavioural beliefs (OR = 1.93, 95% CI [1.09, 3.40]) and normative beliefs (OR = 2.09, 95% CI [1.16, 3.76]) were associated with initial use after multiplicity correction. These associations were attenuated and non-significant in the sensitivity cohort. Control beliefs, attitudes, and behavioural intention showed no consistent associations with initial use. No personal determinant was associated with documentation intensity. The nursing practice environment was not independently associated with either outcome. **Conclusions:** Initial ND use was associated with work exposure and, in the primary cohort, with behavioural and normative beliefs. Baseline individual and organisational factors did not explain documentation intensity, supporting a distinction between initiation and subsequent extent of use.

## 1. Introduction

Nursing diagnoses (NDs) represent the cornerstone of the nursing process, providing a standardised clinical judgement about patients’ responses to health conditions and life processes that guides the selection of appropriate nursing interventions and expected outcomes [1]. Nursing diagnoses make nurses’ clinical judgement explicit, enabling individualised care planning, facilitating continuity of care, improving communication among healthcare professionals, and increasing the visibility of nursing contributions to patient care [2]. Furthermore, documenting nursing diagnoses in a standardised way creates valuable clinical data for monitoring and improving nursing practice [3].

Despite these well-recognised benefits, the implementation of nursing diagnoses remains inconsistent across healthcare settings. Previous studies have reported substantial variability in their adoption, even in organisations where standardised nursing languages and electronic health records are available [3,4,5,6]. The persistence of this implementation gap suggests that introducing diagnostic classifications alone is insufficient to change clinical practice, highlighting the importance of understanding the factors that influence nurses’ documentation behaviour [7].

Research investigating determinants of NDs use has identified both individual and organisational influences. At the individual level, nurses’ knowledge, attitudes toward nursing diagnoses, perceived usefulness, and educational preparation have all been associated with greater and more effective use of nursing diagnoses in clinical practice [8]. At the organisational level, the quality of the nursing practice environment has also emerged as an important determinant. Bozzetti et al. [9] found that the quality of the practice environment was strongly associated with nurses’ approaches to nursing diagnoses. Nurses who perceived a more supportive work environment were more likely to belong to the profile characterised by greater use of nursing diagnoses, whereas those working in less favourable environments were more likely to exhibit negative approaches. However, findings remain heterogeneous, and relatively few studies have examined these determinants within an explicit behavioural theory [10,11].

The Theory of Planned Behaviour (TPB) is one of the most widely used behavioural theories for explaining and predicting professional behaviours [12,13]. In its classical formulation, behaviour is primarily predicted by behavioural intention and perceived behavioural control. Behavioural intention is, in turn, shaped by attitude towards the behaviour, subjective norm, and perceived behavioural control. These proximal constructs are grounded in corresponding belief systems: behavioural beliefs underpin attitudes by reflecting expected consequences of the behaviour; normative beliefs concern the expectations or approval of salient referents and underpin subjective norm; and control beliefs concern factors perceived to facilitate or impede performance and underpin perceived behavioural control [14]. Although the TPB has been successfully applied to explain a wide range of healthcare professionals’ behaviours, its application to the implementation of nursing diagnoses in routine clinical practice remains limited. Furthermore, existing evidence is largely derived from cross-sectional studies, limiting understanding of whether these individual and organisational determinants are associated with the actual implementation of nursing diagnoses over time. In addition, previous studies have typically examined individual and organisational factors separately and have rarely integrated multiple data sources to simultaneously account for nurses’ beliefs, organisational characteristics, and contextual features of the clinical environment that may influence documentation behaviour [11].

Therefore, this study aimed to longitudinally examine the associations of relevant TPB-informed belief domains, attitudes towards NDs, and behavioural intention, together with the nursing practice environment, with subsequent initial ND use and documentation intensity in routine clinical practice. Although the selection of constructs was theory-informed, no directional hypotheses were specified a priori for the associations between individual constructs and either outcome. Accordingly, the construct-specific analyses were exploratory with respect to the direction and magnitude of these prospective associations.

## 2. Materials and Methods

### 2.1. Study Design

Baseline questionnaire data were collected from mid-August to early September 2024. Electronic nursing diagnosis documentation was activated in October, and follow-up continued through December. The study was reported in accordance with the Strengthening the Reporting of Observational Studies in Epidemiology (STROBE) guidelines for cohort studies [15].

### 2.2. Setting

The study was conducted within a single public healthcare organisation in Northern Italy, with the 14 participating adult inpatient clinical units distributed across two hospital sites. All participating units operated according to a broadly comparable functional nursing care delivery model, in which nursing activities were predominantly organised through the allocation of functions and tasks during each shift. Before implementation, NDs were not routinely available for documentation in the electronic nursing record of the participating units. The baseline questionnaire was administered before activation of the ND documentation functionality.

Nursing documentation was introduced across all participating units through a centrally coordinated implementation process. Electronic functionality enabling ND documentation was activated across the 14 units within the same implementation window, representing the beginning of routine electronic ND documentation in these settings. All nurses had access to the same electronic functionality and documentation workflow, which followed a common sequence from nursing assessment to nursing diagnosis and subsequently to nursing interventions. Before go-live, all nurses were required to attend a three-hour in-person theoretical training session focused on the nursing process and the conceptual use of nursing diagnoses. This was subsequently complemented by a one-hour in-person session based on clinical case studies aimed at applying the nursing process to documentation practice. Training was delivered by two PhD-prepared professionals, one of whom had more than 10 years of experience in education in NDs and tutoring. Before implementation, nurse managers attended dedicated meetings in which the project objectives, implementation process, and expected documentation workflow were presented. No formal unit-level implementation champions were designated. During the first month after activation, on-site technical support was provided by two information technology engineers to address issues related to use of the electronic documentation system. Leadership engagement additionally included leadership walkarounds during the implementation period, although these were not delivered according to a standardised unit-level schedule. Consistency across units was promoted through the common implementation window, mandatory training programme, identical electronic documentation functionality and workflow, availability of technical support, and the shared functional nursing care delivery model. The implementation sequence was deliberately structured so that baseline data collection preceded the educational intervention. After completion of the baseline questionnaire, all nurses underwent the mandatory training programme, consisting of the theoretical session and the subsequent case-based workshop. All nurses included in the analytical cohort completed both components before the electronic ND documentation functionality was activated for routine clinical use. However, implementation was not assumed to be identical across units, as local leadership practices, workflow organisation, staffing, clinical activity, and informal peer support could still vary.

### 2.3. Participants

The baseline source population comprised all registered nurses assigned to the 14 participating units at the time of the baseline survey. Eligibility was determined from the personnel registry, which identified 275 nurses working in the participating units during baseline data collection. All eligible nurses were invited to participate. No probability sampling was performed, as a census-based approach was adopted within the workforce involved in the implementation.

#### 2.3.1. Analytical Cohort

The longitudinal cohort included baseline respondents whose questionnaire records could be linked to follow-up electronic documentation and workforce data and who had valid positive work exposure within the participating units. The primary analytical cohort additionally required continuity of clinical unit assignment throughout follow-up. This criterion ensured correspondence between the practice environment assessed at baseline, the unit in which work exposure occurred, and the subsequent documentation outcome. A broader sensitivity cohort retained nurses who moved between participating units during follow-up, provided that their documentation outcomes and work exposure could be determined. The documentation intensity cohort was nested within the primary cohort and included only nurses who documented at least one ND. The workforce observed in the administrative and electronic-record data during follow-up was dynamic and included nurses entering or moving between units after baseline. It was therefore not used as the denominator for baseline participation.

#### 2.3.2. Sample Size

No formal a priori sample size calculation was performed because the study was designed as a real-world prospective cohort embedded within an organisation-wide implementation of NDs documentation. The target population therefore consisted of the entire finite workforce exposed to the implementation at baseline, and all 275 eligible nurses identified through the personnel registry were invited to participate. No probability sampling or target sample recruitment was undertaken.

### 2.4. Data Sources

The analytical dataset was constructed by linking different data sources.

#### 2.4.1. Baseline Questionnaire

The baseline questionnaire dataset contained nurse-level sociodemographic and professional characteristics, beliefs and attitudes towards NDs, and perceptions of the nursing practice environment. Electronic nursing record data covered the study follow-up and contained nursing documentation events attributable to individual nurse accounts. These events were aggregated at the nurse level to determine use and the total number of NDs documented.

#### 2.4.2. Workforce Data

Administrative workforce data included personnel registries, clinical-unit assignments, programmed roster hours, cumulative worked hours, and changes in assignment during follow-up. These data were used to define baseline eligibility, estimate work exposure, identify movements between units, and construct the primary and sensitivity cohorts.

#### 2.4.3. Hospital Discharge Records

Hospital discharge records included Diagnosis-Related Group (DRG) classifications derived from the ICD-9-CM coding system for patients admitted to the participating units during the study period. These data were aggregated at the ward level and used descriptively to characterise clinical activity and case mix across the participating units. Because DRG data could not be linked to individual nurses or to the specific patients cared for by each nurse, ward-level case-mix indicators were not treated as individual-level exposures and were not entered as covariates in the primary regression models.

Records were deterministically linked using a unique nurse identifier, which was replaced with a study-specific pseudonym before analysis. Clinical-unit identifiers were harmonised across data sources. The resulting nurse-level longitudinal dataset included baseline measures, follow-up documentation outcomes, cumulative work exposure, and unit assignment. Hospital discharge data remained at the unit level and were not attributed to individual nurses.

### 2.5. Measurements

#### 2.5.1. Personal Determinants Related to NDs

The Behavioural Beliefs Scale (BBS) is a five-item instrument assessing nurses’ beliefs about the importance of NDs for improving quality of care, selecting appropriate interventions, promoting professional autonomy, facilitating communication, and preserving the person-centred nature of diagnostic labelling [16]. Items are rated on a five-point Likert scale (ranging from 1 = “not important” to 5 = “very important”). Item scores were averaged, with higher scores indicating more favourable behavioural beliefs toward NDs.

The Normative Beliefs Scale (NBS) comprises four items assessing the perceived importance of approval of ND use by relevant social and professional groups, including patients, nursing colleagues, physicians, and nursing managers [16]. Items are rated on a five-point Likert scale (ranging from 1 = “not important” to 5 = “very important”). Higher scores indicate stronger perceived social endorsement of NDs use. Accordingly, the NBS represents a belief-based measure of the importance attributed to social and professional endorsement.

The Control Beliefs Scale (CBS) comprises four items assessing nurses’ beliefs regarding conditions that facilitate the implementation of NDs in clinical practice, including specific education, electronic nursing documentation supported by clinical decision support systems, professional recognition, and organisational support [16]. Items are rated on a five-point Likert scale (ranging from 1 = “not important” to 5 = “very important”). Higher scores indicate stronger perceived importance of facilitating conditions. Accordingly, the CBS represents a belief-based measure of the perceived importance of facilitating conditions.

The Intention to use NDs Scale contains a single item to measure nurses’ intention to use NDs in clinical practice. The item asks, “What is your intention to use NDs in clinical practice?” The score ranges from 0 (“no intention to use”) to 5 (“strong intention to use”).

The Positions on Nursing Diagnosis Scale (PND) is a 20-item semantic differential instrument measuring nurses’ attitudes toward NDs [17]. Each item consists of two opposing adjectives anchored to a seven-point response scale (ranging from 1 = the negative evaluative pole to 7 = the positive evaluative pole, with 4 representing a neutral position).

The BBS, NBS, and CBS had previously undergone psychometric evaluation supporting unidimensional factorial structures and high internal consistency [16]. Confirmatory analyses showed good model fit for the BBS (CFI = 0.95, RMSEA = 0.022, SRMR = 0.032; ω = 0.97), NBS (CFI = 0.98, RMSEA = 0.014, SRMR = 0.026; ω = 0.96), and CBS (CFI = 0.96, RMSEA = 0.018, SRMR = 0.036; ω = 0.94).

#### 2.5.2. Nursing Practice Environment

The Practice Environment Scale of the Nursing Work Index (PES-NWI) measures nurses’ perceptions of organisational characteristics that facilitate or hinder professional nursing practice [18]. The Italian version used in this study comprises 32 items rated on a four-point Likert scale (ranging from 1 = “strongly disagree” to 4 = “strongly agree”). Higher scores indicate a more favourable nursing practice environment. The instrument comprises five dimensions: Staffing and Resource Adequacy (4 items); Nurse Manager Ability, Leadership, and Support (4 items); Nursing Foundations for Quality of Care (10 items); Collegial Nurse–Physician Relations (8 items); and Nurse Participation in Hospital Affairs (6 items). Overall and dimension scores were calculated as the mean of their constituent items. The overall PES-NWI demonstrated excellent internal consistency (α = 0.962). Internal consistency was good to excellent across its five dimensions, ranging from α = 0.877 to α = 0.933.

#### 2.5.3. Electronic Documentation

The observed outcome was the number of nursing diagnoses (NDs) documented by each nurse in the electronic nursing record during the follow-up period. Access to the electronic clinical record required individual authentication through the Italian Public Digital Identity System, allowing for documentation events to be linked to individual user accounts. Nursing diagnoses could not be entered on behalf of another staff member and were recorded under the authenticated account of the nurse who performed the documentation action. Once entered, an ND could not be subsequently modified by another staff member. Consequently, each documentation event was uniquely attributable to the nurse who originally entered the diagnosis. Documentation events were aggregated at the nurse level over the complete follow-up period. Because absence of use and frequency of use may represent different behavioural processes, documentation behaviour was decomposed into two complementary outcomes. Initial use was defined as documentation of at least one ND during the follow-up period (yes/no). This outcome was intended to identify whether a nurse initiated use of the newly introduced functionality and should not be interpreted as evidence of repeated, persistent, or sustained use. Nurses with no documented NDs were classified as non-users, whereas nurses who documented one or more NDs were classified as initial users. Documentation intensity was defined as the number of NDs documented among nurses who had initiated use and was analysed relative to cumulative work exposure. Nurses with zero documented NDs were therefore excluded from the documentation intensity analysis. The threshold of at least one documented ND was selected to distinguish nurses who engaged with the newly available functionality from nurses who did not use it. This operational definition should not be interpreted as evidence of sustained, proficient, or clinically appropriate ND use, and documentation intensity should not be interpreted as an indicator of the quality or appropriateness of the documented diagnoses.

#### 2.5.4. Work Exposure

Individual exposure to opportunities for documenting NDs was estimated from administrative records of cumulative hours worked within the participating clinical units during the same follow-up period. Programmed roster hours, cumulative worked hours, and unit assignments were reconciled to estimate the time each nurse was exposed to the documentation system within the study contexts. Nurses without positive valid exposure were excluded from the analytical cohorts. The logarithm of cumulative worked hours was included as an offset in the documentation intensity models. The models therefore estimated rates of ND documentation relative to individual exposure time rather than modelling unadjusted counts alone. Worked hours represented the time during which a nurse had an opportunity to use the electronic documentation functionality.

### 2.6. Strategies to Address Potential Sources of Bias

Several design and analytical strategies were used to address potential sources of bias. First, beliefs, attitudes, behavioural intention, and perceptions of the practice environment were assessed before NDs were implemented in the electronic record. This temporal ordering reduced the possibility that baseline responses were influenced by subsequent experience with the documentation system. Second, associated factors and outcomes were obtained from different data sources. Personal and organisational determinants were self-reported at baseline, whereas use and documentation intensity were derived from EHRs, reducing the risk of common-method and recall bias. Third, the eligible baseline population was defined using the last available personnel registry rather than the dynamic follow-up roster. This distinction prevented nurses who entered the participating units after baseline from being incorrectly classified as baseline non-respondents. The numbers eligible, participating, linked, and included in each analytical cohort were reported separately. Fourth, all participating units introduced ND documentation within the same organisational implementation and operated according to a comparable functional nursing care delivery model. This provided broadly similar structural opportunities for use, although differences in leadership, local workflow, patient volume, and case mix could remain. Fifth, differences in individual opportunity to use the documentation system were explicitly accounted for using cumulative worked hours. Because use was defined as documenting at least one ND, log-transformed cumulative worked hours were included as a covariate in the use models to account for differential opportunity to perform the behaviour at least once. In documentation intensity models, cumulative worked hours were incorporated as a log offset so that positive ND counts were modelled relative to individual exposure time. Unit-level clustering and residual between-unit heterogeneity were addressed through random intercepts for clinical unit. Sixth, the primary cohort required continuity of clinical-unit assignment, improving correspondence between the practice environment reported at baseline and the clinical context in which follow-up behaviour was observed. A broader sensitivity cohort retained nurses who changed participating units, allowing for the influence of this restriction to be evaluated. Potential confounding was addressed through both design and analytical strategies. Baseline behavioural and organisational measures were collected before implementation, the primary cohort required continuity of clinical-unit assignment, differential work exposure was accounted for analytically, and clinical-unit clustering and residual between-unit heterogeneity were modelled through random intercepts. Ward-level hospital activity and DRG-based case-mix indicators were used for contextual description rather than covariate adjustment. Their aggregation at the clinical-unit level, the inability to attribute patient case mix to individual nurses, and the limited number of participating units precluded reliable estimation of unit-level case-mix effects within the nurse-level models. The PES-NWI-adjusted models were intended specifically to evaluate whether associations with individual-level determinants persisted after accounting for the perceived practice environment and should not be interpreted as fully confounder-adjusted causal models. Given the limited number of non-users, extensive multivariable adjustment for all available individual characteristics was not undertaken to avoid overparameterisation.

### 2.7. Statistical Analyses

Descriptive statistics were used to characterise study variables. Continuous variables were summarised using means (M) and standard deviations (SD) or medians (Me) and interquartile ranges [IQRs], according to their distributions. Categorical variables were summarised using frequencies and percentages. The study protocol and overarching analytical framework were defined a priori. The selection of behavioural and organisational constructs was guided by the theoretical framework and previous literature; however, no directional hypotheses were prespecified for their associations with initial ND use or documentation intensity. Construct-specific analyses were therefore considered exploratory with respect to the direction and magnitude of the observed associations. In particular, the analysis was planned to account for the expected excess of zero documentation counts and the skewed distribution of positive counts. The exact distributional specification was finalised after examination of the observed outcome distribution and model diagnostics. Although a zero-inflated count model had initially been anticipated, the observed data supported a two-part approach that separately modelled use and documentation intensity, allowing for the two behavioural processes to be estimated explicitly while maintaining model parsimony. The BBS, NBS, CBS, PND, behavioural intention, and PES-NWI scores were standardised as z scores before applying the additional restrictions defining the primary and sensitivity cohorts. Coefficients for these variables therefore represent the association corresponding to a one-SD increase in each predictor and are expressed on the same metric across analytical cohorts. Cumulative worked hours were log-transformed to account for their distribution and, for use analyses, standardised within each analytical cohort. The distribution of documentation outcomes included nurses with no documented NDs and an overdispersed distribution of positive counts among users. A two-part mixed-effects strategy was therefore used to model use and documentation intensity as separate behavioural components. Use was analysed using mixed-effects logistic regression. Because the probability of documenting at least one ND depended partly on the duration of opportunity to use the system, standardised log-transformed cumulative worked hours were included as a continuous covariate in all use models. Results are reported as odds ratios (ORs) with 95% confidence intervals (CIs). Documentation intensity among users was analysed using mixed-effects zero-truncated negative binomial regression with an NB2 variance structure. Zero truncation reflected the exclusion of nurses with no documented diagnoses from this component. The logarithm of cumulative worked hours was included as an offset so that documentation frequency was modelled relative to individual exposure time. Results are reported as incidence rate ratios (IRRs) with 95% CIs. All models included a random intercept for clinical unit to account for clustering of nurses within participating units and residual between-unit heterogeneity. BBS, NBS, CBS, PND, and behavioural intention were examined in separate construct-specific models. This approach preserved the theoretical interpretation of each determinant and limited overparameterisation given the number of non-users.

For each personal determinant, two model specifications were estimated. For use, Model 1 included the personal determinant, cumulative work exposure, and the clinical-unit random intercept; Model 2 additionally included the overall PES-NWI score. For documentation intensity, Model 1 included the personal determinant, the worked-hours offset, and the clinical-unit random intercept; Model 2 additionally included the overall PES-NWI score. Accordingly, use models are referred to as work exposure-adjusted and work exposure- plus PES-NWI-adjusted, whereas documentation intensity models are distinguished according to the inclusion or exclusion of the PES-NWI. The overall PES-NWI score and each of its five dimensions were also examined as potential determinants of use and documentation intensity. The overall score and individual dimensions were entered into separate models to avoid collinearity between the aggregate score and its constituent dimensions. Use models examining the PES-NWI were adjusted for cumulative work exposure, whereas documentation intensity models retained the worked hours offset. The incremental contribution of the overall practice environment score was evaluated by comparing corresponding nested models with and without the PES-NWI using Akaike’s information criterion (AIC) and likelihood ratio tests (LRTs). Model adequacy was assessed through convergence and singularity checks, AIC, log-likelihood, ward-level random intercept variance, and marginal R^2^. Conditional R^2^ and intraclass correlation coefficients (ICCs) were additionally reported for mixed-effects logistic use models. For zero-truncated negative binomial models, conditional R^2^ and ICCs were not reported because the distribution-specific variance required for their estimation could not be interpreted reliably. Model comparisons were performed using identical sets of complete observations. To address multiplicity, the Benjamini–Hochberg false discovery rate (FDR) procedure was applied separately within each family of four personal determinants and within each family of five practice environment dimensions. Corrections were conducted separately by outcome and model specification. Behavioural intention was evaluated separately from the four determinant scales, consistent with its distinct theoretical role within the TPB. The overall PES-NWI score constituted a single prespecified test and was not included in the dimension-level correction.

Primary analyses were conducted using complete observations for the variables required by each model, and no statistical imputation was performed. Missingness was assessed for all baseline associated factors, practice environment measures, outcome variables, and exposure variables included in the analytical models. No missing data were present for these variables in the primary analytical cohort. Consequently, all use models included 144 nurses, of whom 122 were users, and all documentation intensity models included the 122 users. A sensitivity analysis repeated the same modelling strategy in the broader cohort that retained nurses who moved between participating units. To explore potential non-response bias, baseline respondents were compared with non-respondents using administrative characteristics available independently of questionnaire participation. The comparison was restricted to nurses whose baseline personnel registry information could be linked within the integrated dataset and included employment duration, employment fraction, full-time employment status, and contract type. Differences were summarised using absolute standardised differences.

Analyses were performed in R 4.5.0 using the glmmTMB [19] package for mixed-effects models, broom.mixed [20] for extraction and presentation of model estimates, and psych [21] for reliability and descriptive psychometric analyses.

## 3. Results

**Figure 1:**
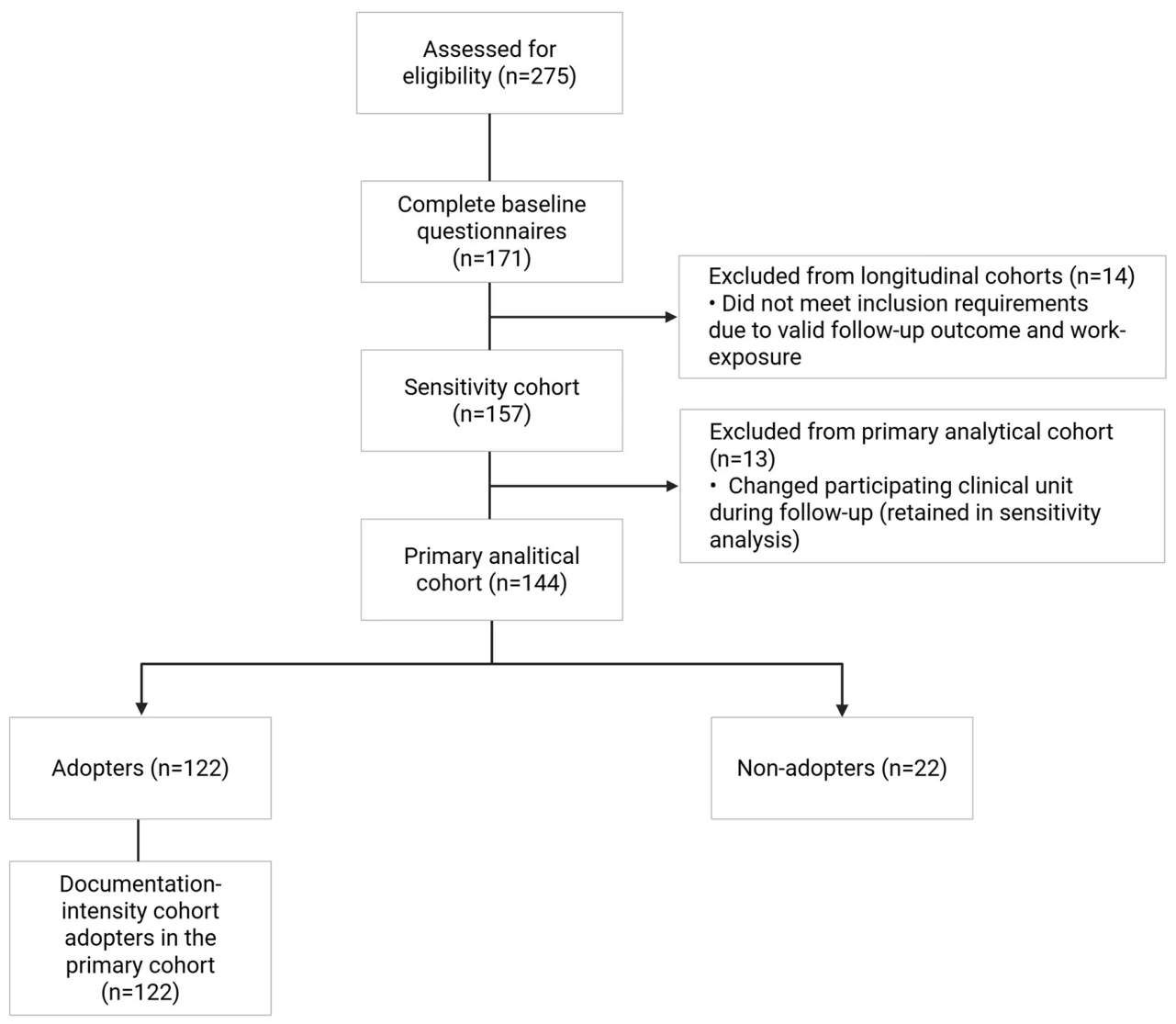
The baseline source population comprised 275 nurses assigned to the 14 participating clinical units. Of these, 171 completed the baseline questionnaire. Fourteen baseline respondents were excluded from longitudinal analyses because they did not meet the minimum eligibility requirements for a valid follow-up documentation outcome and/or positive valid work exposure during follow-up. This yielded a broader sensitivity cohort of 157 nurses. Thirteen nurses who changed participating clinical unit during follow-up were retained in the sensitivity analysis but excluded from the primary analytical cohort, which required continuity of unit assignment. The primary cohort therefore comprised 144 nurses. Of these, 122 documented at least one nursing diagnosis and contributed to the documentation intensity analysis, whereas all 144 contributed to the use analysis.

Participant flow and the construction of the analytical cohorts are shown in Figure 1.

Characteristics of the primary analytical cohort (n = 144), including baseline measures, follow-up work exposure, and ND documentation outcomes, are presented in Table 1.

The 144 nurses in the primary analytical cohort were distributed across 14 adult inpatient clinical units, with variability across units in workforce exposure, patient activity, length of stay, and ND documentation. All 14 units contributed to the nurse-level analyses. DRG-based case-mix indicators were available for 13 units; the Community Hospital was excluded from these comparisons because its activity was not classified under the same DRG tariff system. Detailed clinical-unit characteristics, including workforce exposure, documentation activity, hospital activity, length of stay, and DRG-based case mix, are reported in Table S9.

### 3.1. Personal Determinants and ND Use

**Figure 2:**
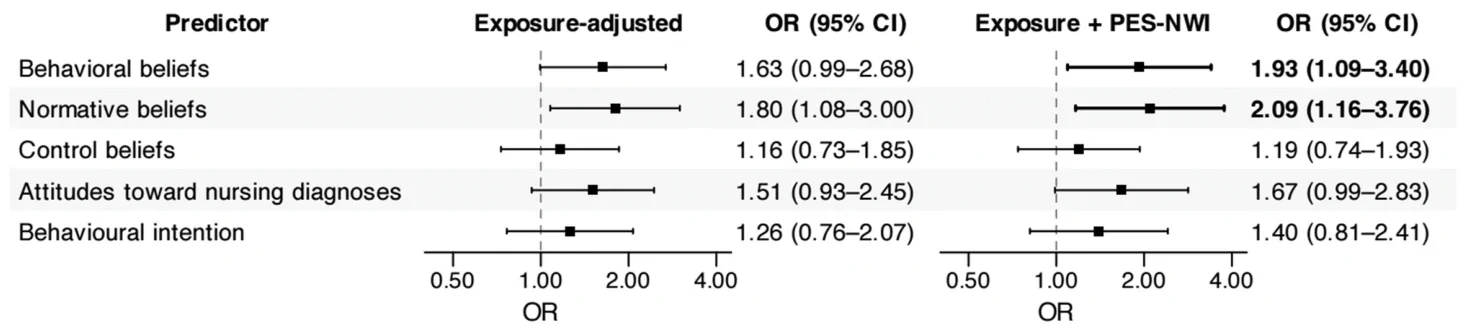
Associations between baseline personal determinants and subsequent ND use. Points and horizontal lines represent odds ratios (ORs) and 95% confidence intervals (CIs) associated with a one-standard-deviation increase in each predictor. Estimates were obtained from separate mixed-effects logistic regression models including a random intercept for study ward. Work exposure-adjusted models did not include the Practice Environment Scale of the Nursing Work Index (PES-NWI), whereas work exposure- plus PES-NWI-adjusted models additionally included the overall PES-NWI score. Bold estimates indicate statistical significance after Benjamini–Hochberg correction. CI = confidence interval; OR = odds ratio; PES-NWI = Practice Environment Scale of the Nursing Work Index.

In the primary cohort, nurses who documented at least one ND worked a median of 590.3 h [IQR 541.9–626.8] during follow-up, compared with 557.7 h [481.9–606.5] among non-users. The absolute standardised difference was 0.52. In a mixed-effects logistic model including the clinical-unit random intercept, greater cumulative work exposure was associated with higher odds of use (OR = 1.71 per one-standard-deviation increase in log-transformed worked hours, 95% CI [1.12, 2.60], *p* = 0.013). After adjustment for work exposure, normative beliefs showed a positive association with use before correction for multiple testing (OR = 1.80, 95% CI [1.08, 3.00], *p* = 0.025), whereas the estimate for behavioural beliefs was of similar direction but less precise (OR = 1.63, 95% CI [0.99, 2.68], *p* = 0.053). Neither association met the Benjamini–Hochberg-adjusted significance criterion in this model specification. Control beliefs, attitudes towards NDs, and behavioural intention were not associated with use. When the overall PES-NWI score was additionally included, behavioural beliefs were associated with higher odds of use (OR = 1.93, 95% CI [1.09, 3.40], *p* = 0.023), as were normative beliefs (OR = 2.09, 95% CI [1.16, 3.76], *p* = 0.014). Both associations remained significant after Benjamini–Hochberg correction (adjusted *p* = 0.047 for both). Attitudes towards NDs showed a positive but less precise estimate (OR = 1.67, 95% CI [0.99, 2.83], *p* = 0.055; adjusted *p* = 0.073). Control beliefs (OR = 1.19, 95% CI [0.74, 1.93]) and behavioural intention (OR = 1.40, 95% CI [0.81, 2.41]) remained unrelated to use (Figure 2).

### 3.2. Personal Determinants and Documentation Intensity

**Figure 3:**
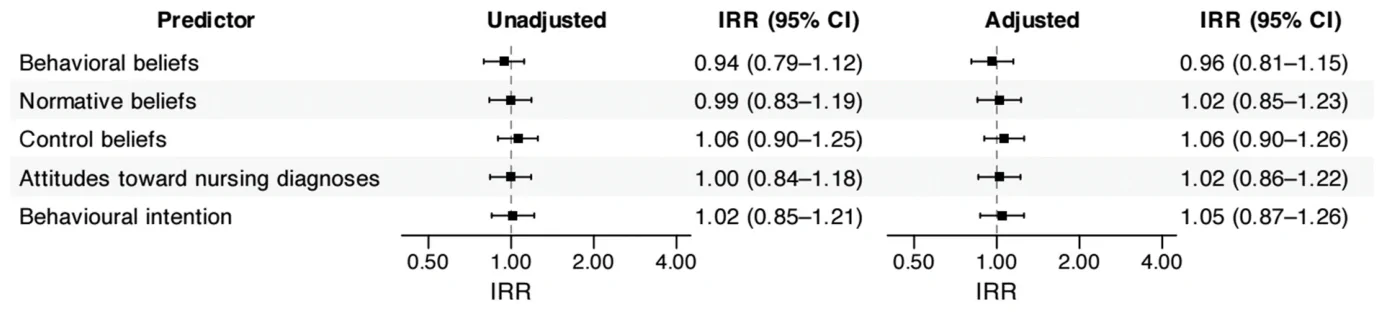
Associations between baseline personal determinants and ND documentation intensity among users. Points and horizontal lines represent incidence rate ratios (IRRs) and 95% confidence intervals (CIs) associated with a one-standard-deviation increase in each predictor. Estimates were obtained from separate mixed-effects zero-truncated negative binomial models including a random intercept for study ward and the logarithm of worked hours as an offset. Models without the Practice Environment Scale of the Nursing Work Index (PES-NWI) included the worked-hours offset and clinical-unit random intercept; models including the PES-NWI additionally included the overall PES-NWI score. Bold estimates indicate statistical significance after Benjamini–Hochberg correction. CI = confidence interval; IRR = incidence rate ratio.

Among the 122 nurses who documented at least one ND, none of the personal determinants was associated with documentation intensity. Estimates were close to the null in both model specifications. In models without adjustment for the PES-NWI, IRRs were 0.94 (95% CI [0.79, 1.12]) for behavioural beliefs, 0.99 (95% CI [0.83, 1.19]) for normative beliefs, 1.06 (95% CI [0.90, 1.25]) for control beliefs, and 1.00 (95% CI [0.84, 1.18]) for attitudes towards NDs. None met the multiplicity-adjusted significance criterion. The estimates remained essentially unchanged after inclusion of the overall PES-NWI score, with IRRs ranging from 0.96 to 1.06 and all confidence intervals including 1. Behavioural intention was likewise unrelated to documentation intensity, both before (IRR = 1.02, 95% CI [0.85, 1.21]) and after adjustment for the PES-NWI (IRR = 1.05, 95% CI [0.87, 1.26]). Thus, neither the belief and attitudinal constructs nor baseline behavioural intention explained variation in documentation frequency once NDs had been adopted (Figure 3).

### 3.3. Nursing Practice Environment and Implementation Outcomes

**Figure 4:**
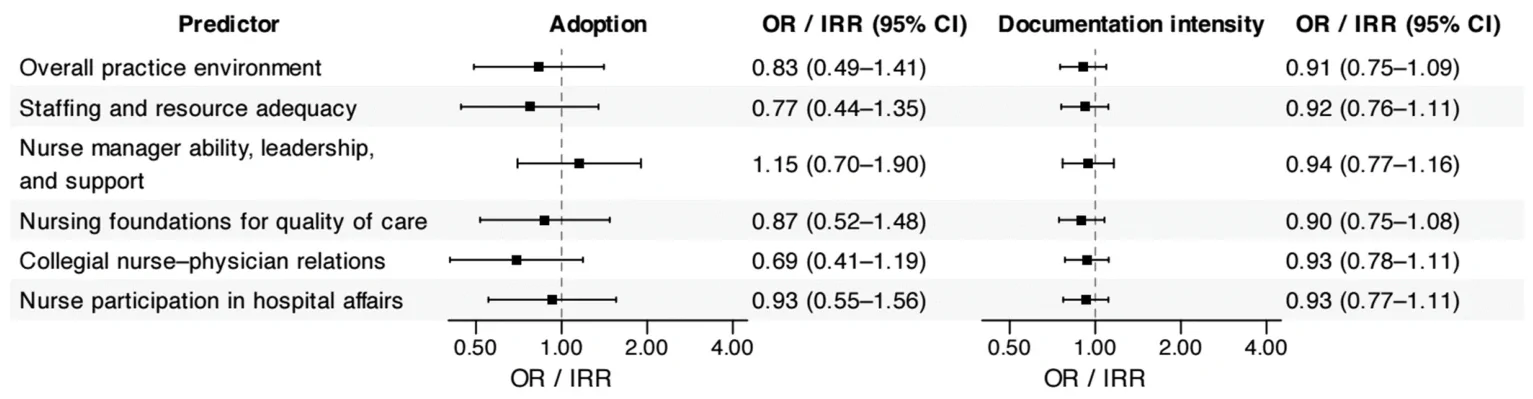
Associations of the overall nursing practice environment and its dimensions with initial ND use and documentation intensity. Points and horizontal lines represent odds ratios (ORs) for use and incidence rate ratios (IRRs) for documentation intensity, with 95% confidence intervals (CIs), associated with a one-standard-deviation increase in each practice environment measure. Each row represents a separate mixed-effects model with a random intercept for study ward. Documentation intensity models additionally included the logarithm of worked hours as an offset. The overall PES-NWI score was evaluated as a single prespecified test.

Neither the overall nursing practice environment nor any of the five PES-NWI dimensions was independently associated with ND use after accounting for cumulative work exposure. For the overall PES-NWI score, the OR for use was 0.83 (95% CI [0.49, 1.41], *p* = 0.493). Dimension-specific estimates ranged from OR = 0.69 for Collegial Nurse–Physician Relations to OR = 1.15 for Nurse Manager Ability, Leadership, and Support, with no association meeting the Benjamini–Hochberg-adjusted significance criterion. Similarly, no evidence of an association was observed between the practice environment and documentation intensity among users. The overall PES-NWI score was not associated with documentation intensity (IRR = 0.91, 95% CI [0.75, 1.09], *p* = 0.305), and dimension-specific IRRs ranged from 0.90 to 0.94. None of the five dimensions met the multiplicity-adjusted significance criterion (Figure 4).

#### Incremental Contribution of the Practice Environment

In the primary cohort, adding the overall PES-NWI score to models that already accounted for cumulative work exposure did not improve model fit for any of the personal determinants. For use, changes in AIC were small (ΔAIC ranging from −0.15 to 1.40), and all likelihood ratio tests were non-significant (*p* = 0.143–0.440). Similarly, inclusion of the PES-NWI did not improve documentation intensity models (ΔAIC = 0.77–1.28; likelihood ratio *p* = 0.267–0.395). Thus, in the primary cohort, there was no evidence that the overall practice environment provided incremental explanatory value beyond the individual determinants and work exposure. In the sensitivity cohort, results were less uniform. Adding the PES-NWI improved fit for the use models including behavioural beliefs (ΔAIC = −2.12; likelihood ratio *p* = 0.042) and normative beliefs (ΔAIC = −1.97; *p* = 0.046). Smaller AIC reductions were observed for attitudes toward NDs and behavioural intention, although the corresponding likelihood ratio tests did not reach statistical significance. No improvement was observed for documentation intensity models. These findings suggest some model-specific incremental contribution of the perceived practice environment in the broader cohort, although this pattern was not observed in the primary cohort and was not consistent across determinants or outcomes.

### 3.4. Sensitivity Analyses

The broader sensitivity cohort comprised 157 nurses, of whom 132 documented at least one ND. Differential work exposure was again evident: users worked a median of 594.0 h [IQR 542.7–627.2], compared with 564.0 h [481.9–607.8] among non-users. The absolute standardised difference was 0.47. Greater cumulative work exposure remained associated with higher odds of use (OR = 1.61 per one-standard-deviation increase in log-transformed worked hours, 95% CI [1.10, 2.36], *p* = 0.014). Associations between personal determinants and use were weaker than those observed in the primary cohort. In the work exposure-adjusted models, behavioural beliefs (OR = 1.29, 95% CI [0.81, 2.04]), normative beliefs (OR = 1.38, 95% CI [0.87, 2.20]), and attitudes towards NDs (OR = 1.25, 95% CI [0.79, 1.97]) retained positive point estimates, but none was statistically significant. Control beliefs and behavioural intention were likewise unrelated to use. After additional adjustment for the overall PES-NWI score, the largest estimates remained those for behavioural beliefs (OR = 1.62, 95% CI [0.95, 2.77]) and normative beliefs (OR = 1.68, 95% CI [0.98, 2.88]); however, neither association met the nominal or Benjamini–Hochberg-adjusted significance criterion. Attitudes towards NDs were also not associated with use (OR = 1.48, 95% CI [0.89, 2.45]), while control beliefs and behavioural intention remained close to the null. The overall PES-NWI score was not independently associated with use in the sensitivity cohort (OR = 0.70, 95% CI [0.42, 1.15]), and none of its five dimensions met the multiplicity-adjusted significance criterion. Documentation intensity analyses remained consistent with the primary cohort, with no evidence of associations for the personal determinants, behavioural intention, or practice environment measures. Among nurses with linkable baseline administrative information, respondents and non-respondents remained closely comparable in employment duration, employment fraction, and full-time employment status. Fixed-term contracts were more frequent among non-respondents than respondents, as reported in Supplementary Table S1.

### 3.5. Model Stability

All 64 fitted models converged successfully, and none showed evidence of singular fit. In the primary cohort personal determinant use models adjusted for work exposure, marginal R^2^ ranged from 0.078 to 0.152, conditional R^2^ from 0.165 to 0.200, and ward-level ICCs from 0.057 to 0.095. After inclusion of the overall PES-NWI score, marginal R^2^ ranged from 0.087 to 0.190, conditional R^2^ from 0.192 to 0.262, and ICCs from 0.087 to 0.121. For documentation intensity models in the primary cohort, marginal R^2^ values remained small, ranging from <0.001 to 0.019 in models without the PES-NWI and from 0.054 to 0.070 after its inclusion. Conditional R^2^ and ICC were not reported for the zero-truncated negative binomial models because the distribution-specific variance required for their estimation could not be interpreted reliably. Model stability was comparable in the sensitivity cohort. For use, marginal R^2^ ranged from 0.059 to 0.085 and conditional R^2^ from 0.146 to 0.153 in work exposure-adjusted models, with ward-level ICCs ranging from 0.071 to 0.094. After addition of the PES-NWI, marginal R^2^ ranged from 0.092 to 0.147, conditional R^2^ from 0.208 to 0.248, and ICCs from 0.111 to 0.133. Marginal R^2^ values for documentation intensity ranged from <0.001 to 0.012 without PES-NWI and from 0.050 to 0.063 after its inclusion. Full model fit statistics, variance components, and model comparisons are reported in the Supplementary Materials.

## 4. Discussion

This longitudinal study examined prospective associations between TPB-informed individual factors, the nursing practice environment, and subsequent ND documentation while accounting for differences in nurses’ opportunity to use the newly introduced electronic functionality. Three findings are central. First, cumulative work exposure was associated with initial ND use, and behavioural and normative beliefs showed the clearest additional prospective signals in the primary cohort, although these associations were sensitive to model specification and were attenuated in the broader sensitivity cohort. Second, none of the measured individual factors, including behavioural intention, was associated with documentation intensity among nurses who had initiated use. Third, neither the overall nursing practice environment nor its dimensions were independently associated with either outcome, although some model-specific incremental contribution of the PES-NWI emerged in the sensitivity cohort.

The association between cumulative work exposure and initial ND use highlights the importance of behavioural opportunity when implementation is examined using routinely recorded clinical data. Nurses who spent more time working within the participating units had more opportunities to encounter situations in which the newly available ND functionality could be used. Accounting for this differential exposure was therefore necessary to distinguish opportunity to perform the behaviour from individual beliefs potentially associated with performing it. After work exposure was taken into account, behavioural and normative beliefs provided the clearest signals for initial ND use. In the primary cohort, both constructs were associated with higher odds of initial use when the overall PES-NWI score was additionally included in the models. However, neither association met the multiplicity-adjusted significance criterion in models accounting for work exposure alone, and both were attenuated in the broader sensitivity cohort. These findings should therefore not be interpreted as establishing behavioural and normative beliefs as invariant predictors of initial use. Rather, they suggest that nurses’ perceptions of the clinical value of NDs and of their endorsement by relevant professional and social referents may contribute to the transition from no recorded use to initial engagement with the documentation practice. This interpretation is broadly consistent with previous nursing research. Behavioural and normative beliefs have been associated with nurses’ attitudes, acceptance, and reported use of nursing diagnoses [10,22], while earlier work specifically examining nurses’ documentation behaviour identified perceived social expectations and behavioural intention as relevant correlates of documentation practices [23].

Control beliefs and attitudes towards NDs did not show robust associations with initial use. Behavioural intention was also not associated with subsequent initial use, despite its proximal theoretical role within the TPB. Previous research among healthcare professionals has generally found behavioural intention to predict subsequent clinical behaviour, while also showing considerable variability in the strength of this relationship and weaker prediction when behaviour is assessed objectively rather than through self-report [24]. In the present study, favourable baseline intention therefore did not appear sufficient, by itself, to distinguish nurses who subsequently initiated ND documentation from those who did not. The findings are compatible with a TPB-informed interpretation of ND implementation. Behavioural, normative, and control beliefs represent belief-based antecedents within the TPB, while attitudes and behavioural intention were assessed more directly. Within this theoretical scope, the results suggest that beliefs concerning the perceived value and professional endorsement of NDs may be more closely related to initial engagement than beliefs concerning facilitating conditions.

A different pattern emerged after nurses had initiated ND documentation. Among users, behavioural beliefs, normative beliefs, control beliefs, attitudes towards NDs, behavioural intention, and perceptions of the nursing practice environment were all unrelated to documentation intensity. This pattern was consistent across model specifications and in the sensitivity cohort. These findings support distinguishing initial use from the extent of subsequent documentation. Implementation frameworks emphasise that different implementation outcomes are conceptually distinct and may be influenced by different mechanisms [25]. In the present study, the factors associated with crossing the threshold from no use to initial use did not explain how frequently NDs were subsequently documented.

Once a new documentation practice has been initiated, its integration into routine work may depend more strongly on factors related to workflow, usability, documentation burden, feedback, and the development of routines. Research on electronic nursing records has identified workflow-related and usability-related characteristics as important contributors to documentation burden [26], while behavioural research among healthcare professionals has highlighted the potential role of planning and habit formation in routinising professional behaviour [27]. Evidence on digital nursing technologies similarly indicates that continued use is shaped by interacting individual, technological, and organisational factors [28]. The absence of factors associated with documentation intensity therefore suggests that the determinants of initial engagement and those supporting routine use may not coincide. Future longitudinal studies should examine these post-use mechanisms more directly, particularly workflow integration, usability, feedback, and the development of stable documentation routines.

Baseline perceptions of the nursing practice environment were not independently associated with either initial ND use or documentation intensity. Neither the overall PES-NWI score nor any of its five dimensions showed a direct association with the study outcomes after accounting for work exposure. In the primary cohort, adding the overall PES-NWI did not improve model fit across the individual-factor models. In the sensitivity cohort, some improvement in fit emerged for models including behavioural and normative beliefs, suggesting a limited and model-specific contribution of the perceived practice environment. The absence of a direct association does not imply that the nursing practice environment is irrelevant to implementation. A substantial study in the literature links favourable nursing work environments with better nurse-, patient-, safety-, and quality-related outcomes [29], while implementation frameworks conceptualise organisational context as shaping the conditions within which implementation occurs [30,31].

This interpretation is particularly relevant in the present study because all participating units introduced ND documentation within a centrally coordinated strategy that included a common implementation window, mandatory theoretical and case-based training, the same electronic functionality and workflow, technical support, and leadership involvement. These shared implementation conditions may have reduced variability in some structural opportunities for using NDs. At the same time, local differences in leadership, staffing, workflow, and clinical activity could still influence how the new documentation practice became embedded in everyday work.

Recent evidence on digital nursing technologies similarly suggests that implementation depends on the interaction of organisational support, leadership, training, workflow, resources, and technological characteristics rather than on any single contextual factor [28]. The PES-NWI may therefore capture important features of the professional nursing environment without fully representing the implementation-specific conditions most closely linked to the uptake of a new electronic documentation practice.

Taken together, the findings suggest that ND implementation should be viewed as a process rather than as a single behavioural endpoint. Initial use appears to be associated with opportunity and, in the primary cohort, with nurses’ perceptions of the value and professional endorsement of the practice, whereas the extent of subsequent documentation was not explained by the baseline individual or organisational measures examined. Implementation strategies may therefore need to address different mechanisms at different stages, combining attention to motivation and opportunity with workflow integration, usability, feedback, and reinforcement of emerging routines [32].

### Strengths and Limitations

This study has several strengths. To our knowledge, it is among the first longitudinal investigations of nursing diagnosis (ND) implementation to combine a theory-informed framework with objectively recorded documentation behaviour derived from electronic nursing records. The longitudinal design established temporal ordering between baseline determinants and subsequent implementation behaviour, while integration of psychological, organisational, workforce, and clinical–administrative data enabled a multidimensional assessment of factors potentially associated with ND use in routine practice. Individual work exposure was explicitly incorporated into the analytical strategy, allowing for differences in nurses’ opportunity to use the newly introduced documentation system to be considered. In addition, multilevel models accounted for the clustering of nurses within clinical units and residual between-unit heterogeneity. Several limitations should nevertheless be considered. First, the study was conducted within a single healthcare organisation, with participating units distributed across two hospital sites, which may limit the generalisability of the findings to settings with different organisational structures, documentation systems, implementation strategies, or nursing care delivery models. Second, the study was informed by the TPB but did not operationalise the complete classical model. The BBS, NBS, and CBS assessed behavioural, normative, and control beliefs, respectively, rather than direct measures of all corresponding proximal TPB constructs. In particular, direct measures of subjective norm and perceived behavioural control were not available. In addition, behavioural intention was assessed using a single item; consequently, internal consistency could not be estimated, and measurement error could not be explicitly modelled. Such measurement error may have attenuated the association between baseline intention and subsequent documentation behaviour and may therefore partly contribute to the absence of an observed association with either implementation outcome. The findings should therefore be interpreted as arising from a TPB-informed assessment of selected belief-based and proximal constructs rather than as a formal test of the complete TPB model, and the observed intention–behaviour gap should be interpreted cautiously. Third, behavioural and attitudinal measures and perceptions of the practice environment were assessed only at baseline, before the mandatory training programme and implementation of electronic ND documentation. Although this temporal ordering allowed for baseline determinants to precede the observed behaviour, these measures should not be regarded as stable characteristics throughout follow-up. Consistent with a TPB-informed perspective, beliefs, attitudes, and intentions may change in response to new information, social influence, perceived facilitating or constraining conditions, and direct behavioural experience. Mandatory training, case-based learning, organisational and technical support, peer interactions, and subsequent experience with the documentation system may therefore have modified the determinants measured at baseline. Because these constructs were not reassessed during follow-up, the study could not examine such changes or their relationship with subsequent implementation behaviour. Fourth, although documentation behaviour was objectively derived from individually authenticated electronic records, the study measured whether and how extensively NDs were documented rather than the quality of the underlying nursing process. Documentation intensity represented the frequency of recorded NDs relative to individual work exposure and should not be interpreted as an indicator of diagnostic quality, clinical accuracy, or appropriateness. The available data did not allow for assessment of whether individual diagnoses were supported by patient assessment findings, appropriately linked with nursing interventions and outcomes, or reflected high-quality clinical reasoning. Consequently, greater documentation intensity may indicate greater use of the electronic functionality without necessarily indicating better-quality nursing documentation or care. Residual confounding also cannot be excluded. Individual characteristics such as previous education or training in NDs, professional experience, and educational preparation may be associated with both baseline behavioural determinants and subsequent documentation behaviour. These variables were not entered simultaneously into the primary construct-specific models because the relatively small number of non-users limited the number of parameters that could be estimated reliably. The multilevel structure should also be interpreted cautiously. Although all fitted models converged successfully, none showed evidence of singular fit, and ward-level ICCs were relatively low, the random intercept variance was estimated from only 14 clinical units and therefore with limited precision. Model-fitting behaviour and variance component patterns were broadly comparable in the sensitivity cohort; however, this diagnostic stability should not be interpreted as evidence that the substantive associations were invariant, as the associations of behavioural and normative beliefs with initial use were attenuated in the broader cohort. Similarly, potentially relevant time-varying factors, including workload, workflow conditions, staffing pressures, and other unit-level characteristics, were not fully captured. Hospital discharge and DRG data were available only at the clinical-unit level, preventing direct attribution of patient allocation, individual workload, or case complexity to specific nurses. Although cumulative worked hours provided an individual measure of opportunity to use the documentation system, it could not capture the number or complexity of clinical situations in which ND documentation was actually indicated. Finally, despite the prospective design, temporal ordering of exposures and outcomes, adjustment for differential work exposure, and multilevel analytical strategy, the observational nature of the study does not support causal inference. Associations should therefore be interpreted as prospective relationships rather than evidence that the measured determinants caused subsequent ND use or documentation behaviour. A further limitation concerns the measurement of opportunity for ND documentation. Cumulative worked hours were used to represent individual exposure time and were incorporated as an offset in documentation intensity models. However, worked hours do not directly measure the number of clinical situations in which an ND could appropriately have been formulated and documented. Such opportunities may have varied across clinical units according to patient volume, case mix, length of stay, workflow, staffing organisation, and local documentation practices. Considerable between-unit variability in ND documentation rates was observed, and although clinical-unit random intercepts were included to account for clustering and residual heterogeneity, they may not have captured all differences in underlying documentation opportunity. The available data did not permit attribution of patient encounters, individual patient load, case complexity, or the number of potentially eligible diagnostic episodes to specific nurses. Documentation intensity estimates should therefore be interpreted as rates relative to time worked rather than rates per true clinical opportunity.

## 5. Conclusions

Behavioural and normative beliefs showed the clearest prospective associations with initial ND use in the primary cohort after accounting for work exposure and the perceived practice environment, although these associations were attenuated in the sensitivity analysis. No consistent associations were observed for control beliefs, attitudes, behavioural intention, or documentation intensity. The findings support distinguishing initial use from the extent and quality of subsequent use. Neither outcome should be interpreted as evidence of sustained implementation or higher-quality nursing practice. Future studies should examine persistence, changes in beliefs over time, and the clinical quality and coherence of ND documentation.

## Figures and Tables

**Table 1:** Nurse characteristics, baseline measures, and follow-up exposure and documentation.

Characteristic	Primary Analytical Cohort (n = 144)
**Sociodemographic and professional characteristics**	
Age, years M (SD)	35.4 (11.6)
Gender n (%)	
Female	116 (80.6%)
Male	28 (19.4%)
Nursing experience, years Me [IQR]	7.0 [2.5, 17.3]
Experience in the current clinical unit, years Me [IQR]	3.0 [1.5, 7.0]
Educational qualification n (%)	
Bachelor’s Degree	29 (20.1%)
Postgraduate	115 (79.9%)
Previous education on NDs n (%)	
Never	27 (18.8%)
Addressed NDs	85 (59.0%)
Highly focused	32 (22.2%)
**Measures**	
**Behavioural and attitudinal measures M (SD)**	
Behavioural Beliefs Scale (BBS)	3.24 (1.09)
Normative Beliefs Scale (NBS)	3.14 (1.17)
Control Beliefs Scale (CBS)	3.92 (1.00)
Positions on Nursing Diagnosis Scale (PND)	4.40 (1.29)
Intention Scale	3.29 (1.20)
**Practice Environment Scale–Nursing Work Index (PES-NWI) M (SD)**	2.31 (0.63)
Staffing and Resource Adequacy (SRA)	2.01 (0.79)
Nurse Manager Ability, Leadership, and Support (NMALS)	2.76 (0.87)
Nursing Foundations for Quality of Care (NFQC)	2.39 (0.63)
Collegial Nurse–Physician Relations (CNPR)	2.26 (0.77)
Nurse Participation in Hospital Affairs (NPHA)	2.13 (0.72)
**Follow-up exposure and documentation**	
Worked hours during follow-up Me [IQR]	588.9 [531.4, 623.1]
Documented at least one ND n (%)	122 (84.7%)
Number of NDs, overall cohort Me [IQR]	20.0 [7.8, 41.5]
Number of NDs among users (n = 122) Me [IQR]	24.5 [13.0, 49.2]
NDs per 1000 worked hours among users (n = 122) Me [IQR]	43.0 [23.3, 87.0]

Data are presented as mean (standard deviation), median [interquartile range], or n (%), as appropriate. Percentages for categorical variables are based on non-missing observations. Scale scores are reported in their original metric. BBS = Behavioural Beliefs Scale; CBS = Control Beliefs Scale; NBS = Normative Beliefs Scale; PES-NWI = Practice Environment Scale of the Nursing Work Index; PND = Positions on Nursing Diagnosis Scale.

## Data Availability

The data presented in this study are available on request from the corresponding author due to privacy restrictions.

## Supplementary Materials

The following supporting information can be downloaded at https://www.mdpi.com/article/10.3390/nursrep16090321/s1, Figure S1: Work exposure-adjusted associations of baseline personal determinants and behavioural intention with subsequent nursing diagnosis use in the primary and sensitivity cohorts; Figure S2: Associations of baseline personal determinants and behavioural intention with subsequent nursing diagnosis use after adjustment for work exposure and the overall PES-NWI score in the primary and sensitivity cohorts; Table S1: Comparison of baseline respondents and non-respondents using available administrative characteristics; Table S2: Distribution of cumulative work exposure according to nursing diagnosis use status and association between work exposure and use; Table S3: Complete primary cohort mixed-effects logistic regression models for nursing diagnosis use; Table S4: Complete primary cohort zero-truncated negative binomial models for nursing diagnosis documentation intensity among users; Table S5: Associations of the overall PES-NWI score and its five dimensions with nursing diagnosis use and documentation intensity in the primary cohort; Table S6: Incremental contribution of the overall PES-NWI score to individual determinant and behavioural intention models; Table S7: Sensitivity cohort associations of individual determinants and behavioural intention with nursing diagnosis use and documentation intensity; Table S8: Model fit, random effect, and diagnostic statistics for the revised primary and sensitivity models; Table S9: Clinical-unit characteristics, nursing diagnosis documentation activity, hospital activity, and DRG-based case-mix indicators.

## Public Involvement Statement

No public involvement in any aspect of this research.

## Guidelines and Standards Statement

This manuscript was prepared in accordance with the Strengthening the Reporting of Observational Studies in Epidemiology (STROBE) Statement for reporting observational research.

## Abbreviations

The following abbreviations are used in this manuscript:

AIC	Akaike Information Criterion
BBS	Behavioural Beliefs Scale
CBS	Control Beliefs Scale
CI	Confidence Interval
CNPR	Collegial Nurse–Physician Relations
DRG	Diagnosis-Related Group
EHR	Electronic Health Record
FDR	False Discovery Rate
ICC	Intraclass Correlation Coefficient
ICD-9-CM	International Classification of Diseases, Ninth Revision, Clinical Modification
IQR	Interquartile Range
IRR	Incidence Rate Ratio
LRT	Likelihood Ratio Test
NBS	Normative Beliefs Scale
ND	Nursing Diagnosis
NDs	Nursing Diagnoses
NFQC	Nursing Foundations for Quality of Care
NMALS	Nurse Manager Ability, Leadership, and Support
NPHA	Nurse Participation in Hospital Affairs
OR	Odds Ratio
PES-NWI	Practice Environment Scale of the Nursing Work Index
PND	Positions on Nursing Diagnosis Scale
SD	Standard Deviation
SRA	Staffing and Resource Adequacy
TPB	Theory of Planned Behaviour
