# Supplementary material for: Prospective Associations of Behavioural and Organisational Factors with Initial Nursing Diagnosis Use: A Longitudinal Study
Source: Nurs Rep. 2026 Sep 4;16(9):321. doi: 10.3390/nursrep16090321 (PMC13610475; doi:10.3390/nursrep16090321)
Supplement: Supplementary file 1 [file nursrep-16-00321-s001.zip › nursrep-4503941-supplementary.pdf]

Supplementary Table S1. Comparison of baseline respondents and non-respondents using available administrative characteristics.

Characteristic	Respondents (n = 171)	Non-respondents with linked baseline administrative data (n = 95)	Absolute standardized difference
Employment duration, years, mean (SD)	8.96 (10.99)	8.72 (11.26)	0.022
Employment fraction, %, mean (SD)	98.54 (6.49)	98.59 (6.29)	0.009
Full-time employment, n (%)	161 (94.2)	90 (94.7)	0.026
Fixed-term contract, n (%)	4 (2.3)	8 (8.4)	0.272

Note. Administrative characteristics were evaluated among the 266 nurses for whom baseline personnel-registry information could be linked within the integrated study dataset (171 respondents and 95 non-respondents). Baseline personnel-registry information could not be linked for nine of the 104 non-respondents. Standardized differences are presented as absolute values. Employment duration was calculated from the administrative date of hire to 26 August 2024. Questionnaire-derived demographic, professional, belief, attitude, and practice-environment measures were unavailable for non-respondents.

Supplementary Table S2. Distribution of cumulative work exposure according to nursing diagnosis use status and association between work exposure and use.

Panel A. Distribution of cumulative worked hours by adoption status

Cohort	Adoption status	n	Worked hours, mean (SD)	Worked hours, median [IQR]	Range	Absolute standardized difference
Primary cohort	Non-adopters	22	516.8 (149.5)	557.7 [481.9, 606.5]	131.6–678.2	—
Primary cohort	Adopters	122	578.7 (79.8)	590.3 [541.9, 626.8]	149.3–717.2	0.517
Sensitivity cohort	Non-adopters	25	525.6 (142.2)	564.0 [482.5, 607.9]	131.6–678.2	—
Sensitivity cohort	Adopters	132	579.6 (78.4)	594.4 [542.8, 627.4]	149.3–717.2	0.471

Panel B. Association between cumulative work exposure and nursing-diagnosis adoption

Cohort	Work-exposure OR (95% CI)	p-value
Primary cohort	1.71 [1.12, 2.60]	0.013
Sensitivity cohort	1.61 [1.10, 2.36]	0.014

Note. Work exposure was derived from cumulative worked hours during follow-up. Absolute standardized differences compare adopters with non-adopters within each cohort. Panel B reports mixed-effects logistic regression models including a random intercept for study ward; the odds ratio represents a one-standard-deviation increase in log-transformed cumulative worked hours. IQR = interquartile range; OR = odds ratio.

Supplementary Table S3. Complete primary cohort mixed-effects logistic regression models for nursing diagnosis use

Predictor	Model	N	Adopters, n	Predictor OR (95% CI)	p	BH-adjusted p	Work exposure OR (95% CI)	Work exposure p	PES-NWI OR (95% CI)	PES-NWI p
Behavioral beliefs	Model 1: work-exposure adjusted	144	122	1.63 [0.99, 2.68]	0.053	0.106	1.63 [1.06, 2.51]	0.026	—	—
Normative beliefs	Model 1: work-exposure adjusted	144	122	1.80 [1.08, 3.00]	0.025	0.099	1.58 [1.04, 2.40]	0.033	—	—
Control beliefs	Model 1: work-exposure adjusted	144	122	1.16 [0.73, 1.85]	0.524	0.524	1.68 [1.10, 2.57]	0.017	—	—
Attitudes toward nursing diagnoses	Model 1: work-exposure adjusted	144	122	1.51 [0.93, 2.45]	0.095	0.127	1.65 [1.07, 2.53]	0.022	—	—
Behavioural intention	Model 1: work-exposure adjusted	144	122	1.26 [0.76, 2.07]	0.367	—	1.65 [1.08, 2.53]	0.020	—	—
Behavioral beliefs	Model 2: work exposure + PES-NWI	144	122	1.93 [1.09, 3.40]	0.023	0.047	1.69 [1.09, 2.63]	0.019	0.65 [0.36, 1.18]	0.156
Normative beliefs	Model 2: work exposure + PES-NWI	144	122	2.09 [1.16, 3.76]	0.014	0.047	1.63 [1.06, 2.49]	0.025	0.66 [0.36, 1.20]	0.173
Control beliefs	Model 2: work exposure + PES-NWI	144	122	1.19 [0.74, 1.93]	0.468	0.468	1.72 [1.12, 2.65]	0.014	0.81 [0.48, 1.38]	0.445
Attitudes toward nursing diagnoses	Model 2: work exposure + PES-NWI	144	122	1.67 [0.99, 2.83]	0.055	0.073	1.70 [1.10, 2.61]	0.017	0.71 [0.40, 1.25]	0.234
Behavioural intention	Model 2: work exposure + PES-NWI	144	122	1.40 [0.81, 2.41]	0.226	—	1.70 [1.11, 2.62]	0.016	0.73 [0.41, 1.31]	0.298

Note. Effect estimates are odds ratios (ORs) and 95% confidence intervals (CIs). Predictor estimates represent a one-standard-deviation increase in each baseline determinant. Model 1 included the individual predictor, standardized log-transformed cumulative worked hours, and a random intercept for study ward. Model 2 additionally included the overall PES-NWI score. Work-exposure ORs correspond to a one-standard-deviation increase in log-transformed worked hours. Benjamini-Hochberg adjustment was applied separately to the four prespecified belief and attitudinal constructs (BBS, NBS, CBS, and PND) within each model specification; behavioural intention (INT) was evaluated separately. PES-NWI = Practice Environment Scale of the Nursing Work Index.

Supplementary Table S4. Complete primary cohort zero-truncated negative binomial models for nursing diagnosis documentation intensity among users.

Predictor	Model	N adopters	Predictor IRR (95% CI)	p	BH-adjusted p	PES-NWI IRR (95% CI)	PES-NWI p
Behavioral beliefs	Model 1	122	0.94 [0.79, 1.12]	0.485	0.955	—	—
Normative beliefs	Model 1	122	0.99 [0.83, 1.19]	0.944	0.955	—	—
Control beliefs	Model 1	122	1.06 [0.90, 1.25]	0.505	0.955	—	—
Attitudes toward nursing diagnoses	Model 1	122	1.00 [0.84, 1.18]	0.955	0.955	—	—
Behavioural intention	Model 1	122	1.02 [0.85, 1.21]	0.866	—	—	—
Behavioral beliefs	Model 2 + PES-NWI	122	0.96 [0.81, 1.15]	0.674	0.825	0.92 [0.76, 1.12]	0.392
Normative beliefs	Model 2 + PES-NWI	122	1.02 [0.85, 1.23]	0.825	0.825	0.90 [0.74, 1.09]	0.295
Control beliefs	Model 2 + PES-NWI	122	1.06 [0.90, 1.26]	0.469	0.825	0.90 [0.75, 1.09]	0.288
Attitudes toward nursing diagnoses	Model 2 + PES-NWI	122	1.02 [0.86, 1.22]	0.811	0.825	0.90 [0.74, 1.09]	0.293
Behavioural intention	Model 2 + PES-NWI	122	1.05 [0.87, 1.26]	0.636	—	0.90 [0.74, 1.09]	0.263

Note. Effect estimates are incidence rate ratios (IRRs) and 95% confidence intervals (CIs) associated with a one-standard-deviation increase in each predictor. All models included a random intercept for study ward and the logarithm of cumulative worked hours as an offset. Model 2 additionally included the overall PES-NWI score. Benjamini-Hochberg adjustment was applied separately to BBS, NBS, CBS, and PND within each model specification; behavioural intention (INT) was evaluated separately. CI = confidence interval; IRR = incidence rate ratio; PES-NWI = Practice Environment Scale of the Nursing Work Index.

Supplementary Table S5. Associations of the overall PES-NWI score and its five dimensions with nursing diagnosis use and documentation intensity in the primary cohort.

Practice-environment measure	Outcome	N	Effect measure	Estimate (95% CI)	p	BH-adjusted p
Overall practice environment	Adoption	144	OR	0.83 [0.49, 1.41]	0.493	—
Staffing and resource adequacy	Adoption	144	OR	0.77 [0.44, 1.35]	0.364	0.768
Nurse manager ability, leadership, and support	Adoption	144	OR	1.15 [0.70, 1.90]	0.574	0.768
Nursing foundations for quality of care	Adoption	144	OR	0.87 [0.52, 1.48]	0.614	0.768
Collegial nurse–physician relations	Adoption	144	OR	0.69 [0.41, 1.19]	0.183	0.768
Nurse participation in hospital affairs	Adoption	144	OR	0.93 [0.55, 1.56]	0.774	0.774
Overall practice environment	Documentation intensity	122	IRR	0.91 [0.75, 1.09]	0.305	—
Staffing and resource adequacy	Documentation intensity	122	IRR	0.92 [0.76, 1.11]	0.384	0.560
Nurse manager ability, leadership, and support	Documentation intensity	122	IRR	0.94 [0.77, 1.16]	0.585	0.585
Nursing foundations for quality of care	Documentation intensity	122	IRR	0.90 [0.75, 1.08]	0.242	0.560
Collegial nurse–physician relations	Documentation intensity	122	IRR	0.93 [0.78, 1.11]	0.448	0.560
Nurse participation in hospital affairs	Documentation intensity	122	IRR	0.93 [0.77, 1.11]	0.411	0.560

Note. Each row represents a separate mixed-effects model. Adoption models were estimated using logistic regression and included standardized log-transformed cumulative worked hours as a covariate and a random intercept for study ward. Documentation-intensity models were estimated using zero-truncated negative binomial regression with the logarithm of worked hours as an offset and a random intercept for study ward. Benjamini-Hochberg adjustment was applied separately to the five PES-NWI dimension-level tests for each outcome; the overall PES-NWI score was evaluated as a single prespecified test. CI = confidence interval; IRR = incidence rate ratio; OR = odds ratio; PES-NWI = Practice Environment Scale of the Nursing Work Index.

Supplementary Table S6. Incremental contribution of the overall PES-NWI score to individual determinant and behavioural intention models.

Panel A. Primary cohort

Outcome	Predictor	AIC without PES-NWI	AIC with PES-NWI	ΔAIC	LR χ²	LR df	LR p
Adoption	Behavioral beliefs	119.55	119.40	-0.15	2.15	1	0.143
Documentation intensity	Behavioral beliefs	1135.07	1136.35	1.28	0.72	1	0.395
Adoption	Normative beliefs	118.25	118.26	0.00	2.00	1	0.158
Documentation intensity	Normative beliefs	1135.56	1136.48	0.92	1.08	1	0.298
Adoption	Control beliefs	122.87	124.27	1.40	0.60	1	0.440
Documentation intensity	Control beliefs	1135.13	1136.01	0.89	1.11	1	0.291
Adoption	Attitudes toward nursing diagnoses	120.51	121.03	0.52	1.48	1	0.223
Documentation intensity	Attitudes toward nursing diagnoses	1135.56	1136.47	0.91	1.09	1	0.296
Adoption	Behavioural intention	122.46	123.33	0.86	1.14	1	0.286
Documentation intensity	Behavioural intention	1135.53	1136.30	0.77	1.23	1	0.267

Panel B. Sensitivity cohort

Outcome	Predictor	AIC without PES-NWI	AIC with PES-NWI	ΔAIC	LR χ²	LR df	LR p
Adoption	Behavioral beliefs	136.86	134.74	-2.12	4.12	1	0.042
Documentation intensity	Behavioral beliefs	1209.74	1210.81	1.08	0.92	1	0.337
Adoption	Normative beliefs	136.20	134.23	-1.97	3.97	1	0.046
Documentation intensity	Normative beliefs	1210.08	1210.88	0.80	1.20	1	0.272
Adoption	Control beliefs	137.93	137.64	-0.29	2.29	1	0.130
Documentation intensity	Control beliefs	1209.77	1210.42	0.66	1.34	1	0.247
Adoption	Attitudes toward nursing diagnoses	137.10	135.58	-1.51	3.51	1	0.061
Documentation intensity	Attitudes toward nursing diagnoses	1210.06	1210.89	0.83	1.17	1	0.279
Adoption	Behavioural intention	137.70	136.50	-1.20	3.20	1	0.074
Documentation intensity	Behavioural intention	1210.11	1210.84	0.74	1.26	1	0.262

Note. For adoption, the model without PES-NWI already included standardized log-transformed cumulative worked hours and a random intercept for study ward. For documentation intensity, both nested models included the logarithm of worked hours as an offset and a ward random intercept. Negative ΔAIC values indicate lower AIC after adding the overall PES-NWI score. Likelihood-ratio tests compare each model with the corresponding model additionally including the overall PES-NWI score. AIC = Akaike information criterion; LR = likelihood ratio; PES-NWI = Practice Environment Scale of the Nursing Work Index.

Supplementary Table S7. Sensitivity cohort associations of individual determinants and behavioural intention with nursing diagnosis use and documentation intensity.

Outcome	Predictor	N	Adopters, n	Model 1 estimate (95% CI)	Model 1 p	Model 1 BH-adjusted p	Model 2 estimate (95% CI)	Model 2 p	Model 2 BH-adjusted p
Adoption	Behavioral beliefs	157	132	1.29 [0.81, 2.04]	0.284	0.455	1.62 [0.95, 2.77]	0.079	0.158
Adoption	Normative beliefs	157	132	1.38 [0.87, 2.20]	0.176	0.455	1.68 [0.98, 2.88]	0.059	0.158
Adoption	Control beliefs	157	132	1.06 [0.67, 1.66]	0.810	0.810	1.13 [0.70, 1.82]	0.615	0.615
Adoption	Attitudes toward nursing diagnoses	157	132	1.25 [0.79, 1.97]	0.341	0.455	1.48 [0.89, 2.45]	0.130	0.173
Adoption	Behavioural intention	157	132	1.14 [0.71, 1.81]	0.593	—	1.37 [0.81, 2.32]	0.241	—
Documentation intensity	Behavioral beliefs	132	—	0.95 [0.80, 1.13]	0.538	0.846	0.97 [0.81, 1.17]	0.774	0.933
Documentation intensity	Normative beliefs	132	—	0.98 [0.82, 1.17]	0.846	0.846	1.01 [0.84, 1.22]	0.888	0.933
Documentation intensity	Control beliefs	132	—	1.05 [0.89, 1.25]	0.550	0.846	1.06 [0.90, 1.26]	0.488	0.933
Documentation intensity	Attitudes toward nursing diagnoses	132	—	0.98 [0.82, 1.17]	0.813	0.846	1.01 [0.84, 1.21]	0.933	0.933
Documentation intensity	Behavioural intention	132	—	0.99 [0.83, 1.19]	0.910	—	1.02 [0.85, 1.23]	0.821	—

Note. Effect estimates are odds ratios (ORs) for adoption and incidence rate ratios (IRRs) for documentation intensity, with 95% confidence intervals. For adoption, Model 1 included the individual predictor, standardized log-transformed cumulative worked hours, and a random intercept for study ward; Model 2 additionally included the overall PES-NWI score. For documentation intensity, Model 1 included the predictor, ward random intercept, and log worked-hours offset; Model 2 additionally included the overall PES-NWI score. Benjamini-Hochberg adjustment was applied separately to BBS, NBS, CBS, and PND for each outcome and model specification; behavioural intention (INT) was evaluated separately.

Supplementary Table S8. Model fit, random effect, and diagnostic statistics for the revised primary and sensitivity models.

Sample	Model family	Outcome	Specification	Predictor	N	AIC	Log-likelihood	Marginal R²	Conditional R²	Ward variance	ICC	Convergence	Singular fit
Primary	Personal determinant	Adoption	Work-exposure adjusted	Behavioral beliefs	144	119.55	-55.77	0.133	0.199	0.272	0.076	Yes	No
Primary	Personal determinant	Adoption	Work exposure + PES-NWI	Behavioral beliefs	144	119.40	-54.70	0.169	0.262	0.413	0.112	Yes	No
Primary	Personal determinant	Documentation intensity	Without PES-NWI	Behavioral beliefs	122	1135.07	-563.54	0.019	—	0.196	—	Yes	No
Primary	Personal determinant	Documentation intensity	With PES-NWI	Behavioral beliefs	122	1136.35	-563.17	0.055	—	0.184	—	Yes	No
Primary	Personal determinant	Adoption	Work-exposure adjusted	Normative beliefs	144	118.25	-55.13	0.152	0.200	0.197	0.057	Yes	No
Primary	Personal determinant	Adoption	Work exposure + PES-NWI	Normative beliefs	144	118.26	-54.13	0.190	0.260	0.313	0.087	Yes	No
Primary	Personal determinant	Documentation intensity	Without PES-NWI	Normative beliefs	122	1135.56	-563.78	<0.001	—	0.192	—	Yes	No
Primary	Personal determinant	Documentation intensity	With PES-NWI	Normative beliefs	122	1136.48	-563.24	0.055	—	0.178	—	Yes	No
Primary	Personal determinant	Adoption	Work-exposure adjusted	Control beliefs	144	122.87	-57.44	0.078	0.165	0.346	0.095	Yes	No
Primary	Personal determinant	Adoption	Work exposure + PES-NWI	Control beliefs	144	124.27	-57.14	0.087	0.192	0.428	0.115	Yes	No
Primary	Personal determinant	Documentation intensity	Without PES-NWI	Control beliefs	122	1135.13	-563.56	0.019	—	0.195	—	Yes	No
Primary	Personal determinant	Documentation intensity	With PES-NWI	Control beliefs	122	1136.01	-563.01	0.070	—	0.182	—	Yes	No
Primary	Personal determinant	Adoption	Work-exposure adjusted	Attitudes toward nursing diagnoses	144	120.51	-56.26	0.117	0.196	0.320	0.089	Yes	No
Primary	Personal determinant	Adoption	Work exposure + PES-NWI	Attitudes toward nursing diagnoses	144	121.03	-55.52	0.137	0.241	0.452	0.121	Yes	No
Primary	Personal determinant	Documentation intensity	Without PES-NWI	Attitudes toward nursing diagnoses	122	1135.56	-563.78	<0.001	—	0.192	—	Yes	No
Primary	Personal determinant	Documentation intensity	With PES-NWI	Attitudes toward nursing diagnoses	122	1136.47	-563.23	0.054	—	0.177	—	Yes	No
Primary	Personal determinant	Adoption	Work-exposure adjusted	Behavioural intention	144	122.46	-57.23	0.084	0.167	0.326	0.090	Yes	No
Primary	Personal determinant	Adoption	Work exposure + PES-NWI	Behavioural intention	144	123.33	-56.66	0.103	0.211	0.452	0.121	Yes	No
Primary	Personal determinant	Documentation intensity	Without PES-NWI	Behavioural intention	122	1135.53	-563.77	0.001	—	0.192	—	Yes	No
Primary	Personal determinant	Documentation intensity	With PES-NWI	Behavioural intention	122	1136.30	-563.15	0.062	—	0.177	—	Yes	No
Sensitivity	Personal determinant	Adoption	Work-exposure adjusted	Behavioral beliefs	157	136.86	-64.43	0.076	0.152	0.295	0.082	Yes	No
Sensitivity	Personal determinant	Adoption	Work exposure + PES-NWI	Behavioral beliefs	157	134.74	-62.37	0.139	0.248	0.479	0.127	Yes	No
Sensitivity	Personal determinant	Documentation intensity	Without PES-NWI	Behavioral beliefs	132	1209.74	-600.87	0.012	—	0.244	—	Yes	No
Sensitivity	Personal determinant	Documentation intensity	With PES-NWI	Behavioral beliefs	132	1210.81	-600.41	0.050	—	0.224	—	Yes	No
Sensitivity	Personal determinant	Adoption	Work-exposure adjusted	Normative beliefs	157	136.20	-64.10	0.085	0.150	0.251	0.071	Yes	No

Sample	Model family	Outcome	Specification	Predictor	N	AIC	Log-likelihood	Marginal R^2	Conditional R^2	Ward variance	ICC	Convergence	Singular fit
Sensitivity	Personal determinant	Adoption	Work exposure + PES-NWI	Normative beliefs	157	134.23	-62.11	0.147	0.242	0.409	0.111	Yes	No
Sensitivity	Personal determinant	Documentation intensity	Without PES-NWI	Normative beliefs	132	1210.08	-601.04	0.001	—	0.243	—	Yes	No
Sensitivity	Personal determinant	Documentation intensity	With PES-NWI	Normative beliefs	132	1210.88	-600.44	0.051	—	0.221	—	Yes	No
Sensitivity	Personal determinant	Adoption	Work-exposure adjusted	Control beliefs	157	137.93	-64.96	0.059	0.148	0.342	0.094	Yes	No
Sensitivity	Personal determinant	Adoption	Work exposure + PES-NWI	Control beliefs	157	137.64	-63.82	0.092	0.208	0.481	0.128	Yes	No
Sensitivity	Personal determinant	Documentation intensity	Without PES-NWI	Control beliefs	132	1209.77	-600.88	0.012	—	0.246	—	Yes	No
Sensitivity	Personal determinant	Documentation intensity	With PES-NWI	Control beliefs	132	1210.42	-600.21	0.063	—	0.224	—	Yes	No
Sensitivity	Personal determinant	Adoption	Work-exposure adjusted	Attitudes toward nursing diagnoses	157	137.10	-64.55	0.072	0.153	0.314	0.087	Yes	No
Sensitivity	Personal determinant	Adoption	Work exposure + PES-NWI	Attitudes toward nursing diagnoses	157	135.58	-62.79	0.120	0.236	0.497	0.131	Yes	No
Sensitivity	Personal determinant	Documentation intensity	Without PES-NWI	Attitudes toward nursing diagnoses	132	1210.06	-601.03	0.002	—	0.243	—	Yes	No
Sensitivity	Personal determinant	Documentation intensity	With PES-NWI	Attitudes toward nursing diagnoses	132	1210.89	-600.44	0.050	—	0.221	—	Yes	No
Sensitivity	Personal determinant	Adoption	Work-exposure adjusted	Behavioural intention	157	137.70	-64.85	0.062	0.146	0.323	0.089	Yes	No
Sensitivity	Personal determinant	Adoption	Work exposure + PES-NWI	Behavioural intention	157	136.50	-63.25	0.110	0.228	0.503	0.133	Yes	No
Sensitivity	Personal determinant	Documentation intensity	Without PES-NWI	Behavioural intention	132	1210.11	-601.05	<0.001	—	0.242	—	Yes	No
Sensitivity	Personal determinant	Documentation intensity	With PES-NWI	Behavioural intention	132	1210.84	-600.42	0.052	—	0.221	—	Yes	No
Primary	PES-NWI	Adoption	Work-exposure adjusted	Overall PES-NWI	144	122.79	-57.39	0.079	0.206	0.524	0.137	Yes	No
Primary	PES-NWI	Documentation intensity	Worked-hours offset	Overall PES-NWI	122	1134.52	-563.26	0.051	—	0.179	—	Yes	No
Primary	PES-NWI	Adoption	Work-exposure adjusted	Staffing and Resource Adequacy	144	122.42	-57.21	0.086	0.220	0.563	0.146	Yes	No
Primary	PES-NWI	Documentation intensity	Worked-hours offset	Staffing and Resource Adequacy	122	1134.82	-563.41	0.040	—	0.170	—	Yes	No
Primary	PES-NWI	Adoption	Work-exposure adjusted	Nurse Manager Ability, Leadership, and Support	144	122.95	-57.48	0.077	0.177	0.397	0.108	Yes	No
Primary	PES-NWI	Documentation intensity	Worked-hours offset	Nurse Manager Ability, Leadership, and Support	122	1135.26	-563.63	0.019	—	0.179	—	Yes	No
Primary	PES-NWI	Adoption	Work-exposure adjusted	Nursing Foundations for Quality of Care	144	123.01	-57.50	0.076	0.195	0.486	0.129	Yes	No
Primary	PES-NWI	Documentation intensity	Worked-hours offset	Nursing Foundations for Quality of Care	122	1134.22	-563.11	0.066	—	0.176	—	Yes	No

Sample	Model family	Outcome	Specification	Predictor	N	AIC	Log-likelihood	Marginal R^2	Conditional R^2	Ward variance	ICC	Convergence	Singular fit
Primary	PES-NWI	Adoption	Work-exposure adjusted	Collegial Nurse-Physician Relations	144	121.38	-56.69	0.099	0.245	0.635	0.162	Yes	No
Primary	PES-NWI	Documentation intensity	Worked-hours offset	Collegial Nurse-Physician Relations	122	1134.99	-563.50	0.023	—	0.197	—	Yes	No
Primary	PES-NWI	Adoption	Work-exposure adjusted	Nurse Participation in Hospital Affairs	144	123.18	-57.59	0.073	0.187	0.461	0.123	Yes	No
Primary	PES-NWI	Documentation intensity	Worked-hours offset	Nurse Participation in Hospital Affairs	122	1134.89	-563.45	0.032	—	0.182	—	Yes	No
Sensitivity	PES-NWI	Adoption	Work-exposure adjusted	Overall PES-NWI	157	135.89	-63.94	0.089	0.214	0.525	0.138	Yes	No
Sensitivity	PES-NWI	Documentation intensity	Worked-hours offset	Overall PES-NWI	132	1208.90	-600.45	0.049	—	0.222	—	Yes	No
Sensitivity	PES-NWI	Adoption	Work-exposure adjusted	Staffing and Resource Adequacy	157	134.88	-63.44	0.105	0.241	0.590	0.152	Yes	No
Sensitivity	PES-NWI	Documentation intensity	Worked-hours offset	Staffing and Resource Adequacy	132	1209.51	-600.75	0.026	—	0.219	—	Yes	No
Sensitivity	PES-NWI	Adoption	Work-exposure adjusted	Nurse Manager Ability, Leadership, and Support	157	137.98	-64.99	0.059	0.152	0.361	0.099	Yes	No
Sensitivity	PES-NWI	Documentation intensity	Worked-hours offset	Nurse Manager Ability, Leadership, and Support	132	1209.83	-600.92	0.015	—	0.225	—	Yes	No
Sensitivity	PES-NWI	Adoption	Work-exposure adjusted	Nursing Foundations for Quality of Care	157	136.72	-64.36	0.078	0.195	0.481	0.127	Yes	No
Sensitivity	PES-NWI	Documentation intensity	Worked-hours offset	Nursing Foundations for Quality of Care	132	1207.65	-599.82	0.096	—	0.218	—	Yes	No
Sensitivity	PES-NWI	Adoption	Work-exposure adjusted	Collegial Nurse-Physician Relations	157	134.32	-63.16	0.107	0.233	0.539	0.141	Yes	No
Sensitivity	PES-NWI	Documentation intensity	Worked-hours offset	Collegial Nurse-Physician Relations	132	1209.88	-600.94	0.008	—	0.240	—	Yes	No
Sensitivity	PES-NWI	Adoption	Work-exposure adjusted	Nurse Participation in Hospital Affairs	157	136.83	-64.42	0.075	0.194	0.483	0.128	Yes	No
Sensitivity	PES-NWI	Documentation intensity	Worked-hours offset	Nurse Participation in Hospital Affairs	132	1208.93	-600.47	0.045	—	0.229	—	Yes	No

Note. Marginal R^2 represents variance explained by fixed effects, whereas conditional R^2 additionally incorporates the ward-level random effect. Conditional R^2 and ICC are reported for mixed-effects logistic adoption models. For zero-truncated negative binomial documentation-intensity models, conditional R^2 and ICC were not reported because the distribution-specific variance required for their estimation was not considered reliably interpretable. Ward variance refers to the random-intercept variance at study-ward level. All 64 models converged successfully and none showed evidence of singular fit. AIC = Akaike information criterion; ICC = intraclass correlation coefficient; PES-NWI = Practice Environment Scale of the Nursing Work Index.

Supplementary Table S9. Clinical-unit characteristics, nursing diagnosis documentation activity, hospital activity, and DRG-based case-mix indicators.

Clinical unit	Nurses, n	Adopters, n (%)	Worked hours	NDs, n	NDs per 1,000 worked hours	Patient-days, n	Length of stay, Me [IQR], days	Distinct DRGs, n	Dominant DRG, n(%)	Mean tariff value, €
Cardiology	16	10 (62.5%)	8,857	145	16.4	1,978	4.0 [2.0, 6.0]	29	557 – Percutaneous cardiovascular procedures with drug-eluting stent with major cardiovascular diagnosis, 89 (22.9%)	€5,752
General Surgery	10	8 (80.0%)	6,349	280	44.1	3,449	2.0 [1.0, 5.0]	85	494 – Laparoscopic cholecystectomy without common bile duct exploration, without complications or comorbidities, 103 (14.4%)	€3,778
Multispecialty Surgery	2	2 (100.0%)	634	43	67.8	2,565	2.0 [1.0, 4.0]	81	311 – Transurethral procedures without complications or comorbidities, 87 (13.6%)	€3,097
Hematology	11	8 (72.7%)	6,313	92	14.6	1,039	19.0 [9.5, 25.5]	22	492 – Chemotherapy associated with a secondary diagnosis of acute leukemia or involving high-dose chemotherapeutic agents, 9 (19.1%)	€8,771
Infectious Disease	8	8 (100.0%)	4,312	521	120.8	2,029	10.0 [6.0, 20.0]	41	421 – Viral diseases, age > 17 years, 15 (11.6%)	€4,321
Internal Medicine	14	12 (85.7%)	7,919	628	79.3	7,473	12.0 [7.0, 19.0]	86	127 – Heart failure and shock, 72 (14.8%)	€3,949
Emergency Medicine	9	9 (100.0%)	5,487	386	70.4	1,270	5.0 [4.0, 7.0]	68	321 – Kidney and urinary tract infections, age > 17 years, without complications or comorbidities, 18 (8.7%)	€2,542
Nephrology	11	9 (81.8%)	5,970	231	38.7	2,357	9.0 [5.0, 16.0]	22	316 – Renal failure, 110 (53.9%)	€4,181
Neurosurgery	18	16 (88.9%)	9,967	571	57.3	3,014	7.0 [3.0, 14.0]	44	002 – Craniotomy, age > 17 years, without complications or comorbidities, 55 (19.6%)	€9,488
Neurology	10	10 (100.0%)	5,444	435	79.9	1,108	7.0 [5.0, 11.0]	28	014 – Intracranial hemorrhage or cerebral infarction, 52 (42.3%)	€6,387
Orthopedics	15	11 (73.3%)	9,083	285	31.4	2,308	3.0 [2.0, 7.0]	42	544 – Major joint replacement or reattachment of lower extremity, 57 (14.6%)	€4,227
Community Hospital	6	6 (100.0%)	3,424	220	64.3	—	—	—	—	—
Pulmonology	14	13 (92.9%)	8,218	1,008	122.7	3,401	6.0 [4.0, 10.0]	39	087 – Pulmonary edema and respiratory failure, 239 (58.6%)	€4,161
**Overall**	**144**	**122 (84.7%)**	**81,978**	**4,845**	**59.1**	**31,991**	—	—	—	—

Note. Nurse exposure and NDs documentation refer to nurses in the primary analytical cohort. Ward-level activity and case-mix indicators were derived from hospital discharge and DRG data. The dominant DRG is the most frequent DRG within each clinical unit. DRG-based activity and case-mix indicators refer to 13 of the 14 participating units. The Community Hospital contributed to the study cohort and nurse-level analyses but was excluded from DRG-based comparisons because its activity was not classified under the same DRG tariff system. DRG = Diagnosis-Related Group; ND = Nursing Diagnosis; IQR = interquartile range. "—" indicates that the indicator was unavailable or not meaningfully aggregable.
