# Supplementary material for: Prospective Associations of Behavioural and Organisational Factors with Initial Nursing Diagnosis Use: A Longitudinal Study
Source: Nurs Rep. 2026 Sep 4;16(9):321. doi: 10.3390/nursrep16090321 (PMC13610475; doi:10.3390/nursrep16090321)
Supplement: Supplementary file 1 [file nursrep-16-00321-s001.zip › Supplementary Figure S2.pdf]

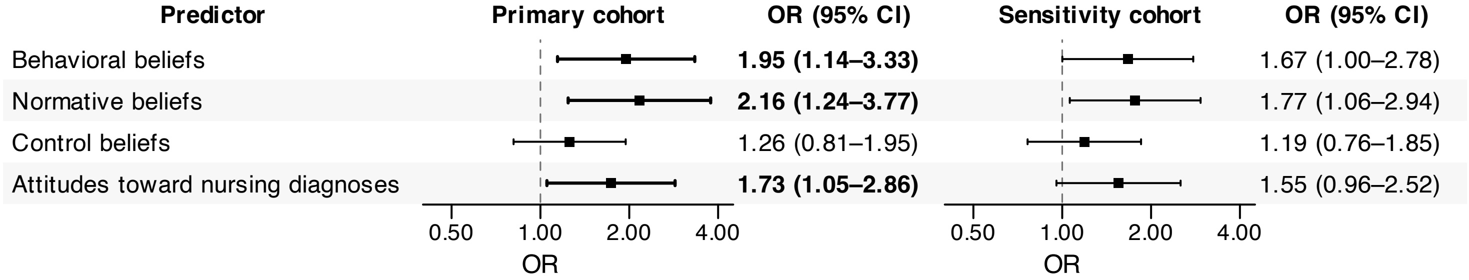

Predictor
Primary cohort
OR (95% CI)
Sensitivity cohort
OR (95% CI)
Behavioral beliefs
1.95 (1.14–3.33)
1.67 (1.00–2.78)
Normative beliefs
2.16 (1.24–3.77)
1.77 (1.06–2.94)
Control beliefs
1.26 (0.81–1.95)
1.19 (0.76–1.85)
Attitudes toward nursing diagnoses
1.73 (1.05–2.86)
1.55 (0.96–2.52)
0.50
1.00
2.00
4.00
OR
0.50
1.00
2.00
4.00
OR
